# T1234: A distortion‐matched structural scan solution to misregistration of high resolution fMRI data

**DOI:** 10.1002/mrm.30480

**Published:** 2025-03-13

**Authors:** Chung Kan, Rüdiger Stirnberg, Marcela Montequin, Omer Faruk Gulban, A. Tyler Morgan, Peter A Bandettini, Laurentius Huber

**Affiliations:** ^1^ NIH Bethesda USA; ^2^ German Center for Neurodegenerative Diseases (DZNE) Bonn Germany; ^3^ CN, FPN, University of Maastricht The Netherlands; ^4^ Brain Innovation Maastricht The Netherlands

**Keywords:** 7 T acquisition, distortion, layer‐fMRI, T_1_ mapping

## Abstract

**Purpose:**

Registration of functional and structural data poses a challenge for high‐resolution fMRI studies at 7 T. This study aims to develop a rapid acquisition method that provides distortion‐matched, artifact‐mitigated structural reference data.

**Methods:**

We introduce an efficient sequence protocol termed T1234, which offers adjustable distortions. This includes data that match distortions of functional data and data that are free of distortions. This approach involves a T_1_‐weighted 2‐inversion 3D‐EPI sequence with four combinations of read and phase encoding directions optimized for high‐resolution fMRI. A forward Bloch model was used for T_1_ quantification and protocol optimization. Fifteen participants were scanned at 7 T using both structural and functional protocols to evaluate the use of T1234.

**Results:**

Results from two protocols are presented. A fast distortion‐free protocol reliably produced whole‐brain segmentations at 0.8 mm isotropic resolution within 3:00–3:40 min. It demonstrates robustness across sessions, participants, and three different 7 T SIEMENS scanners. For a protocol with geometric distortions that matched functional data, T1234 facilitates layer‐specific fMRI signal analysis with enhanced laminar precision.

**Conclusion:**

This structural mapping approach enables precise registration with fMRI data. T1234 has been successfully implemented, validated, and tested, and is now available to users at our center and at over 50 centers worldwide.

## 1 INTRODUCTION

High‐resolution fMRI promises to reveal fine‐scale neural information flow across layers and columns. However, common approaches of high‐resolution fMRI with Cartesian EPI are limited by EPI phase artifacts dominantly from eddy‐current induced trajectory imperfections and B_0_‐related geometrical distortions. A survey by the International Society for Magnetic Resonance in Medicine (ISMRM) brain function study group found that the most limiting factor of high‐resolution fMRI is image registration.^1^ This is despite the fact that researchers commonly invest significant time (≈10 min) in acquiring high‐quality anatomical data with MP2RAGE.^2^

In this study, we build on previous works.^3^, ^4^, ^5^, ^6^, ^7^, ^8^, ^9^, ^10^, ^11^, ^12^ by developing, implementing, characterizing, and validating a novel acquisition method: T1234, involving T_1_‐weighted acquisition with two inversions using a 3D EPI readout in four spatial encoding directions. This sequence provides structural reference images with high anatomical contrast, adjustable geometrical distortions, and mitigated artifacts in 3 to 4 min.

### 1.1 Historical heritage of magnetization‐prepared EPI

Since the advent of EPI‐based fMRI,^13^, ^14^ geometric distortions have been a recognized challenge and have subsequently been addressed through dewarping techniques.^15^ T_1_‐weighted EPI protocols were introduced approximately the same time.^16^, ^17^, ^18^ With 7 T fMRI at higher resolution, Huber et al.^3^ used T_1_‐weighted EPI in first attempts to estimate voxels of upper and deeper layers in the distorted functional space of constraint fMRI slabs. High‐resolution T_1_‐EPI was then combined with more advanced strategies, including slice‐wise inversion time cycling for broader 2D‐EPI coverage,^5^, ^6^ simultaneous multi‐slice readouts,^9^ 3D‐EPI, and multi‐shot readouts.^8^, ^11^, ^19^ Van der Zwaag et al. named their approach T123, referring to T_1_‐imaging with two 3D‐EPIs. The ability of obtaining maps of quantitative estimates of T_1_ can be helpful for fMRI with respect to reproducibility across scanners and vendors. It might allow contextualization of the fMRI results with the participant‐specific cortical laminar myeloarchitecture. Further, T_1_‐EPI developments have combined advanced 3D‐EPI techniques with alternative magnetization transfer‐preparation methods.^4^, ^10^ These advancements, achieving sub‐millimeter resolutions on 7 T scanners, were driven by the goal of facilitating mesoscale layer‐fMRI analyses. However, none of these approaches has yet gained widespread adoption. It is unclear why that is. We suspect that this is largely because of the inherent noise in EPI, which typically demands long acquisition times and averaging. Moreover, some noise sources stem from consistent EPI trajectory imperfections, which cannot be mitigated through averaging alone.

Our work here builds on the method by Stirnberg et al.^8^ They had developed a Look‐Locker inversion‐recovery 3D‐EPI sequence for efficient T_1_ mapping. Here, we use the developed sequence, including the loop structure of the multi‐shot 3D‐EPI. We further optimize this technique by integrating multi‐phase encoding directions,^10^ interleaved dual‐polarity readouts,^11^, ^20^ contrast‐to‐noise ratio (CNR) optimizations by means of TI‐specific flip angles that are larger than in conventional Look‐Locker approaches, and we combined it with a forward Bloch model^16^ for T_1_ quantification. This sequence is tested for two protocols. A distortion‐matched protocol that allows layer segmentation in the functional data without spatial warping and a fast whole brain protocol that can be used with respective analysis for obtaining data with any desired distortions, including distortion‐free data as well as matching distortions to functional data.

## 2 METHODS

We tested the T1234 sequence in 15 MRI sessions across three 7 T scanners: two SIEMENS Terra scanners and one SIEMENS 7 T Plus scanner. MRI data were acquired under the National Institutes of Health‐institutional review board (NIH‐IRB) (93‐M‐0170, ClinicalTrials.gov: NCT00001360).

### 2.1 Acquisition

We used a highly segmented 3D‐EPI sequence with inversion preparation and high resolution readout. The EPI readout is used with multiple combinations of gradient polarities to quantify and correct for geometric distortions and EPI ghosting artifacts.

The use of two polarities of the read gradient allows mitigating typical EPI low spatial frequency artifacts, known as “fuzzy ripples,” which result from imperfections in k‐space trajectories and off‐resonance effects.^19^, ^21^

Two images acquired in reverse phase encoding directions have opposite distortions. The adjustable EPI‐segmentation level and phase bandwidth in T1234 can be matched to the effective echo spacing of the functional data, thereby aligning distortions at the acquisition level. Alternatively, with distortions in opposite directions, researchers can estimate the distortion field and retrospectively synthesize any desired level of geometric warping, similar to the TOPUP method,^22^ here implemented in Analysis of Functional NeuroImages (AFNI).

**FIGURE 1:**
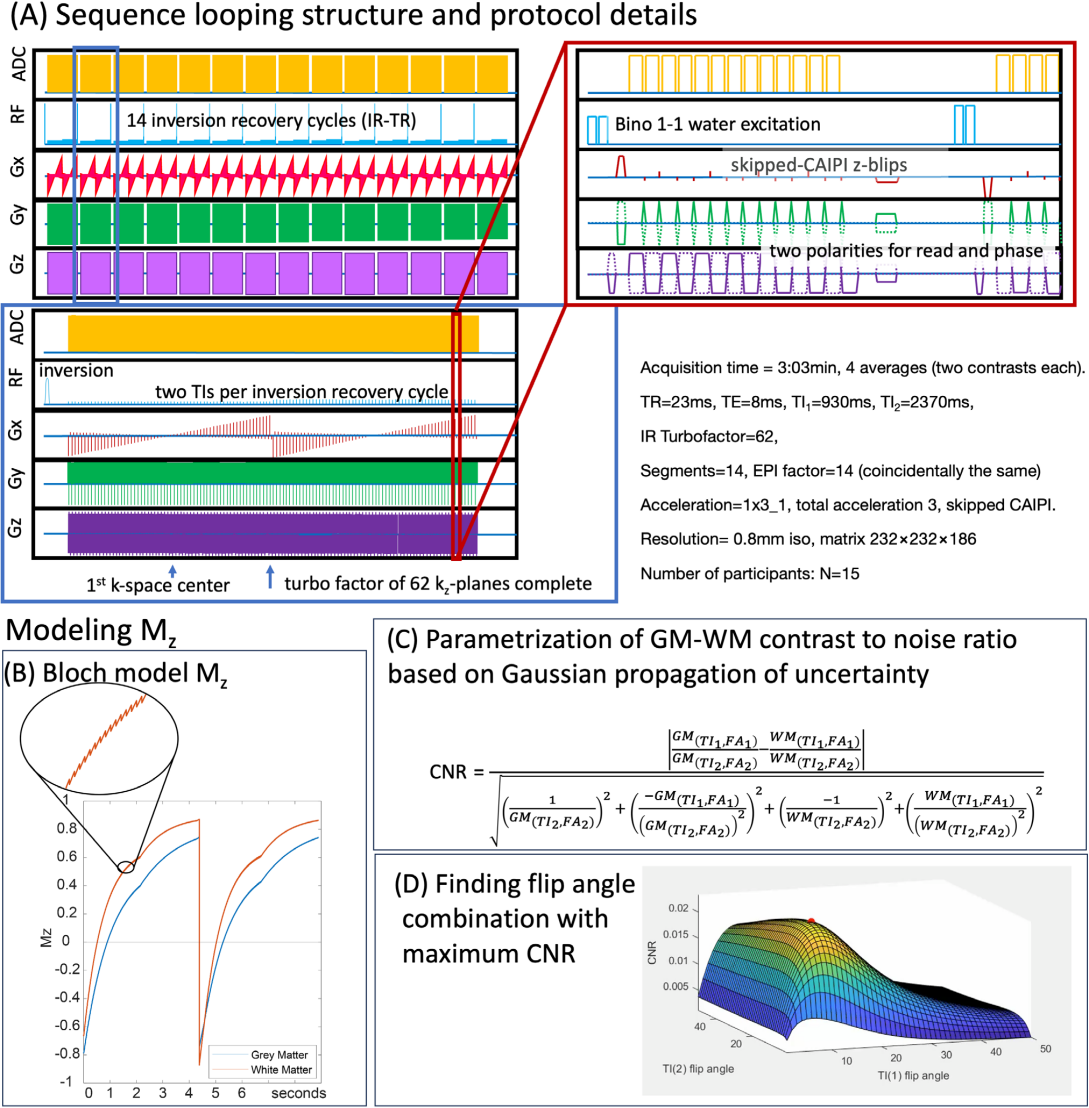
Sequence diagram and corresponding expected magnetization. (A) Individual panels are zoomed‐in sections of one complete volume acquisition of a given read and phase polarity combinations. This illustrates the sequence looping structure across interleaved multi‐shot EPI‐segments, inversion recovery cycles, kz planes, and ky lines. This entire cycle of a volume acquisition is repeated four times, incorporating all combinations of reversed read and phase directions. (B) This panel schematically depicts the Bloch model that represents the expected Mz magnetization in the inversion‐recovery cycle with non‐selective excitation pulses. The saw‐tooth pattern in the zoomed‐in section results from the EPI‐segment‐specific excitation flip angle. The two distinct apparent relaxation EPI‐segments correspond to two different flip angles for the first and second inversion times. (C) Based on this Bloch model, the expected gray matter‐white matter contrast‐to‐noise ratio (CNR) can be parameterized as a Gaussian propagation of uncertainty. Gray matter (GM) and white matter (WM) refer to the signal intensity of the respective issue compartments, here estimated as *M*_x/y_ components right after the excitation pulse. CNR is estimated following the equations in appendix 2 of the MP2RAGE paper.^2^ The contrast is defined as the relative signal difference between GM and WM (in units of −1 to +1) for linear functions, with independent variables (GM signal and WM signal). (D) This panel shows the CNR as a function of the two flip angles for the first and second inversion times, respectively. The local maximum is highlighted with a red dot.

Key protocol parameters across experiments included: 0.8 mm isotropic resolution, skipped‐CAIPI^7^ 1 × 3z1, EPI‐segmentation factor of 14,^8^, ^19^ matrix size of 232 × 232 × 186, and a phase partial Fourier factor of 6/8. We used a relatively high EPI‐segmentation factor to minimize distortions and T_2_*‐blurring. For a complete list of parameters, see: https://github.com/layerfMRI/Sequence_Github/tree/master/T1234 (tag: T1234release). The loop structure of this sequence^8^, ^21^ is illustrated in Figure 1 for the proposed protocol.

We used two approaches to obtaining distortion matched T_1_‐weighted structural data for subsequence layer‐fMRI that we aimed to investigate here.

#### 2.1.1 Retrospectively distorting highly segmented acquisitions to match distortions of functional data

EPI data with opposite phase encoding allows a retrospective estimation of the local distortion field. Knowing the effective echo spacing of the functional data, the T1234 data were deliberately warped to match the distortion of the functional data. Different to conventional TOPUP‐like distortion correction in fMRI, a high EPI‐segmentation factor can be used in T1234 to mitigate common shortcoming of within voxel dephasing contaminations.

A total of 15 participants were scanned with this “retrospective distortion‐matched approach.” The full parameter list is compiled in the protocol repository as: T1234_retrospectively_distrotion_matched_oblique. IR related parameters: TI1/TI2/TE/TRshot/TRIR = 930/2368/8/23/3097 ms, Flip angle (FA) = 10°.

#### 2.1.2 Acquiring structural data with matching distortions

The bandwidth, EPI‐segmentation, and undersampling factor R of the T1234 protocol can be adjusted to match the effective echo spacing of the functional data. This means that T1234 is inherently distortion‐matched to the functional data.

Five participants were scanned with this “acquisition distortion‐matched approach,” two of these participants also performed a functional task with visual stimulation in the same session. In these experiments, we kept the effective echo spacing identical to that of a conventional fMRI protocol for 7 T at standard resolutions.^23^

The most relevant parameters are: resolution 0.8 mm, effective echo spacing 0.35 ms, and total acquisition 3:30 min. The full parameter list is compiled in the protocol repository as: T1234_acquisition_distortion_matched.

The Inversion recovery (IR) related parameters are: TI1/TI2/TE/TR_shot_/TR_IR_ = 1378/3793/26/78/5050 ms, FA = 10°.

The acquisition parameters of the functional protocol are listed in the repository https://github.com/layerfMRI/Sequence_Github/blob/master/T1234/functional_protocol.pdf (tag: T1234release). Most important parameters are: *SS‐SI VASO*,^3^ TR_vol_/TR_pair_/TE/TI1/TI2 = 1690/4336/25/1345/3044 ms, 0.75 mm iso voxels, *R* = 3.

The temporal stability of the sequence was assessed with a temporal Signal to Noise Ratio (tSNR), 2 experiment containing 40 volumes at volume TRs of 52 s and acquisition times of 35 min using one single phase encoding direction (subjects n = 2), using a protocol like for “retrospective distortion‐matching protocol.” tSNR was estimated after motion correction for individual read directions and then averaged for both read directions.

To explore T1234's capabilities for even higher resolutions, we investigated data acquired in another study at 0.47 mm resolutions^12^ (protocol parameters: https://github.com/layerfMRI/Sequence_Github/tree/master/Terra_protocolls/0p47mm, tag: T1234release).

As reference, MP2RAGE^2^ was acquired with an imaging protocol commonly used in layer fMRI studies: no B_1_^+^ correction, TR = 4.5 s, resolution 0.8 mm, FAs = 4/5 (see MP2RAGE.pdf in Github reference).

### 2.2 Reconstruction and analysis

Complex‐valued averaging was performed after parallel image reconstruction and coil combination using IcePat.^24^ IcePat is a vendor implementation of GRAPPA that allows reconstruction of 3D k‐space data with phase encode undersampling in two directions with CAIPI FOV shifting between EPI‐segments (also known as hexagonal sampling). Nyquist ghost correction was carried out using the vendor's standard two‐parameter implementation.^25^, ^26^, ^27^ The same navigator and GRAPPA reference data were used for both EPI read polarities. For each phase encoding direction polarity, a different set of phase navigators and GRAPPA reference data was acquired and used.

Motion correction of complex‐valued image space data was performed using ANTS.^28^ Specifically, we averaged the real and imaginary parts of the complex valued signal across images that were acquired with inverse read gradient polarities. Because the EPI ghost is having an opposite phase across read gradient polarities, this averaging cancels the ghost and retains the signal. Finally, real and imaginary parts were converted to estimate magnitude images as the square root of the sum of squares. Layers were extracted using the Layer‐Toolbox framework (Barilary: https://github.com/marcobarilari/layerfMRI‐toolbox) with LayNii v2.6.0,^29^ FreeSurfer^30^ v7.4.1, and PreSurfer 1f5ad28 as a wrapper for SPM12 tools.^31^ Geometric distortions were estimated and applied as in TOPUP^22^ with the AFNI program 3dQwarp. Specifically, the images of opposing phase gradient polarities were used to estimate a distortion field for the effective echo spacing of the T1234 acquisition. This spatial distortion field was multiplied to match the effective echo spacing of the functional data to obtain a distortion‐matched dataset. In a copy of the data, the inverse of the distortion field was applied to match distortions with a hypothetical echo spacing of zero. This was done to obtain distortion free reference data for comparison.

### 2.3 Bloch simulations for sequence optimization and T_1_ estimation

**FIGURE 2:**
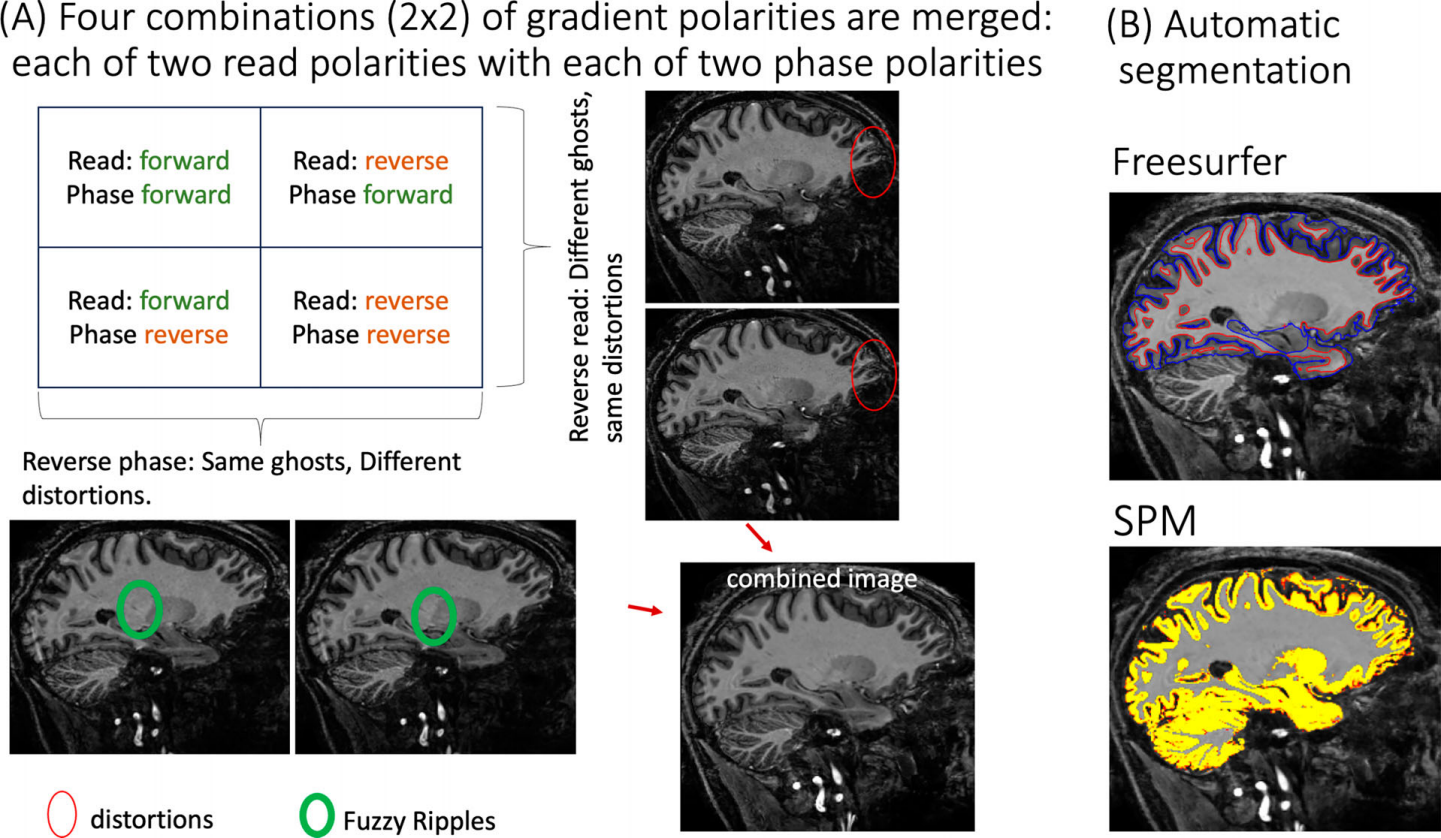
Merging four combinations of read and phase polarity imaging for tissue‐type segmentation. (A) Example image showing artifacts that are mitigated by combining read and phase polarities. The ellipses highlight artifact levels that are benignly inverted by reversing read and phase polarity. Green ellipses indicate “fuzzy ripples”, a low spatial frequency artifact caused by gradient trajectory imperfections at EPI ramp‐sampling corners. For an animated version of this figure that more clearly depicts the fuzzy ripples, see here: (https://github.com/layerfMRI/repository/blob/master/T1234/animation_2.gif. (B) After combining the four images, the data can be processed through mainstream automatic segmentation pipelines. Representative results from FreeSurfer and SPM are shown, respectively. T1234 data in this figure were scanned with protocol parameters as described in the “retrospectively distortion‐matched approach.”

**FIGURE 3:**
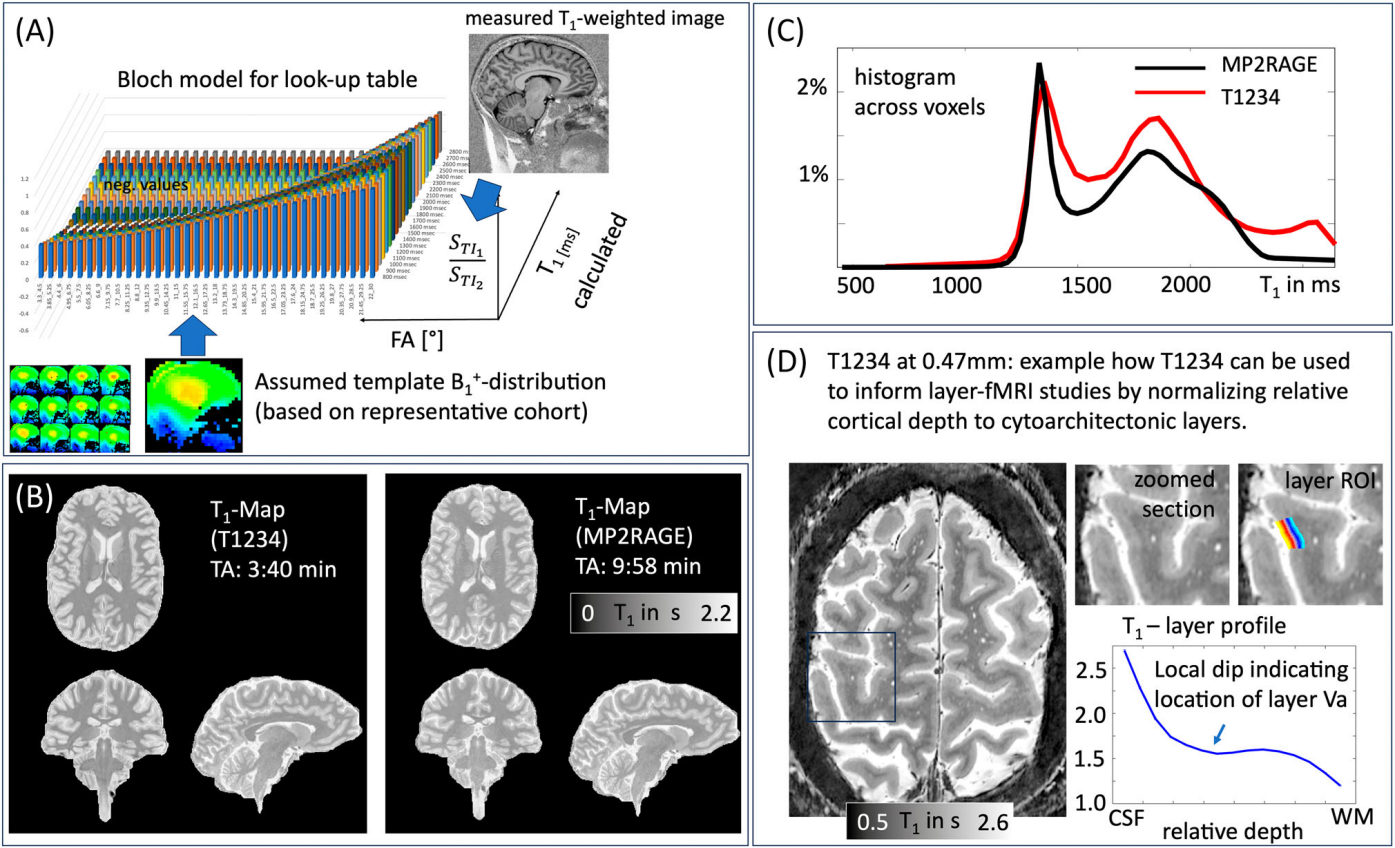
T_1_ quantification for the T1234 approach. (A) The Bloch solver (Figure 1B) is used to generate a lookup table of expected relative MR signal intensities for T_1_ tissue types at both inversion times, depending on TR, flip angle (FA), and the Inversion recovery (IR) looping structure. (B,C) Representative T_1_ map and corresponding histogram generated using the proposed approach, compared to a T_1_ map generated with MP2RAGE. The gray matter (GM) and white matter (WM) peaks are largely identical. (D) Example application of slab‐selective T1234 for layer‐fMRI. At higher spatial resolutions for layer‐specific mapping, T1234 can be used to identify myeloarchitectonic landmarks to calibrate cortical depth to layers. Although lacking a ground truth of the laminar T_1_‐profile, the existence and the location of the dip matches myeloarchitecture compared in previous research.^35^, ^36^ T1234 data in this figure were scanned with protocol parameters as described in the “retrospectively distortion‐matched approach.”

To predict the optimal distribution of variable flip angles across the inversion‐recovery evolution, based on the used loop structure (Figure 1,2), we implemented a forward Bloch simulation. This simulation allowed us to determine the excitation flip angles that provide the maximum CNR between gray matter and white matter (Figure 3). The Bloch solver was also used to generate a lookup table (Figure 3A) that converts relative signal changes to physical units of T_1_ in milliseconds. Given that the protocol used here uses relatively large flip angles (>10°), a simple Look‐Locker analysis approach that assumes small flip angels is not justified. The spin‐history of each readout alters the T_1_‐relaxation curve depending on local B_1_ inhomogeneity‐dependent excitation flip angles. As a first‐order approximation of locally dependent Mz‐saturation, we used a universal low‐resolution template B_1_ map, previously obtained across the cortex in 20 participants with 2 mm MAFI.^32^ Simulation code and analysis scripts are available at: https://github.com/layerfMRI/repository/tree/master/T1234.

## 3 RESULTS

Figure 2 depicts representative results from one participant. The signal maps from the first inversion time show strong gray matter‐white matter (GM‐WM) contrast and good SNR with minimal graininess. However, these images also display conventional EPI artifacts, including geometric distortions and “fuzzy ripples” (Figure 2A). By combining data from multiple read and phase polarity directions, these artifacts can be mitigated, making the images suitable for standard tissue segmentation (Figure 2B).

Figure 3A illustrates how T1234 data can be used to estimate quantitative T_1_‐values. This is done by using a lookup table derived from a forward Bloch model to convert measured signal ratios of TI1 and TI2 into T_1_ values in milliseconds. The resulting T_1_ values are compared with those derived from MP2RAGE (because this is what is typically available for fMRI studies) as maps (Figure 3B) and as histograms (Figure 3C). Slight deviations might arise because of unmodeled magnetization transfer effects during the readout in either sequence, potentially incomplete inversion efficiency, imperfections in the MP2RAGE T_1_ model for relatively short TRs (4.5 s), lack of B_1_^+^ transmit field inhomogeneity correction in MP2RAGE, inaccuracies in flip angle estimation in the T1234 Bloch model based on the universal B_1_ distribution approach used here. More investigations are needed to be sure.

Quantitative T_1_ mapping can be valuable for calibrating geometric cortical layers^33^ to myeloarchitectonic layers,^34^ using the shape of quantitative T_1_ profiles as anatomical landmarks. This is exemplified in Figure 3D with a modified slab‐selective high‐resolution T1234 protocol at 0.47 mm isotropic.

**FIGURE 4:**
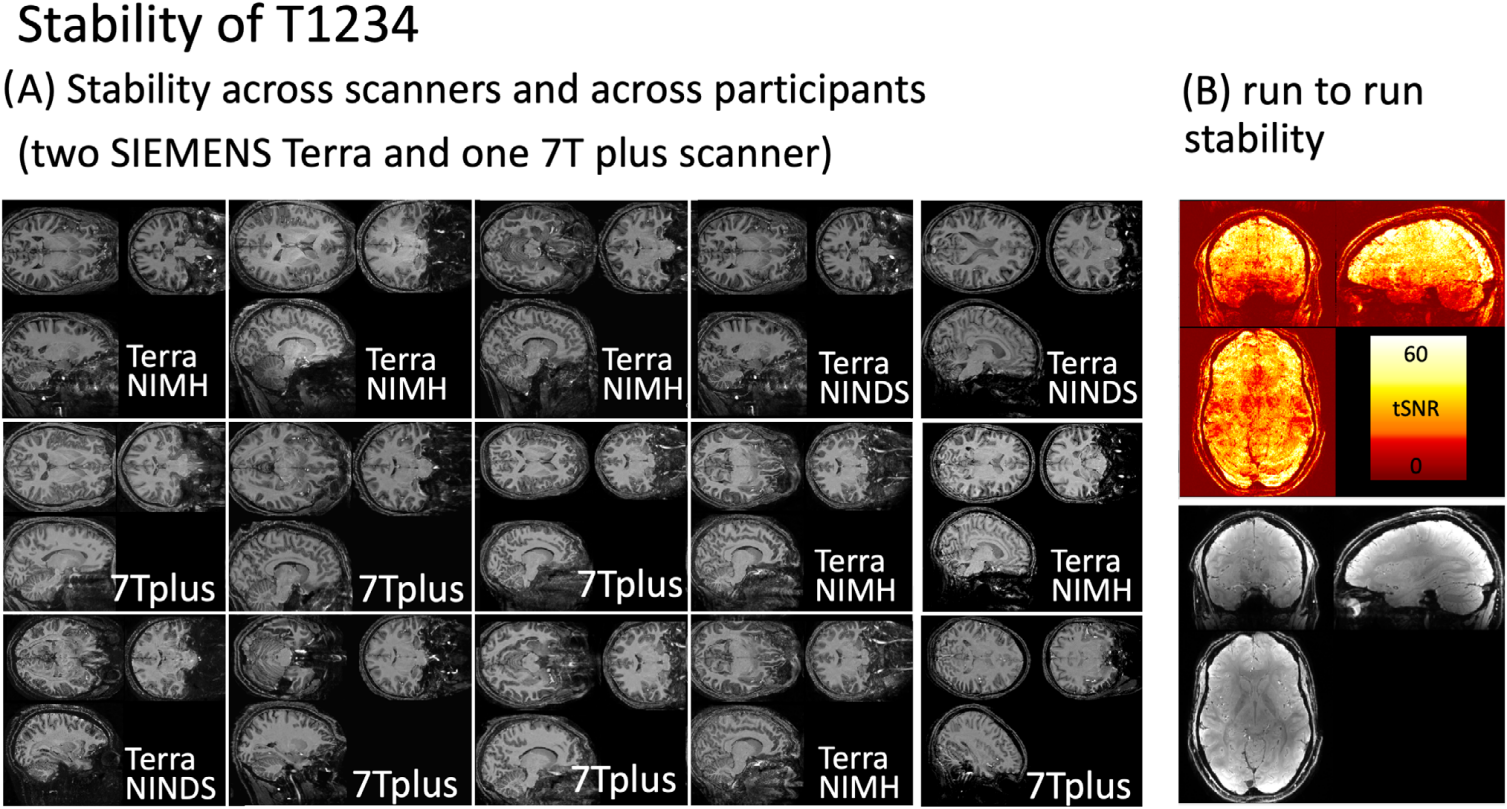
Stability of T1234. (A) Depicts T1234 images across 15 scan sessions, showing repeatable applicability across participants and different scanner models. The protocol reliably provides strong T_1_ contrast. For all of these participants, segmentations could be estimated. These data were acquired with the acquisition protocol that was optimized for potential retrospective distortion matching. Here, the distortion‐free data are shown. (B) Shows run‐to‐run stability of the protocol in the form of temporal Signal to Noise Ratio (tSNR) maps (top), calculated as the mean (bottom) divided by the standard deviation. T1234 data in this figure were scanned with protocol parameters as described in the “retrospectively distortion‐matched approach.” Detailed zoomed in 3D data can be explored in shared nifty data (see data sharing statement).

We found that the tested T1234 protocol can be repeatedly applied across participants, sessions, scanners (Figure 4A), and runs (Figure 4B).

**FIGURE 5:**
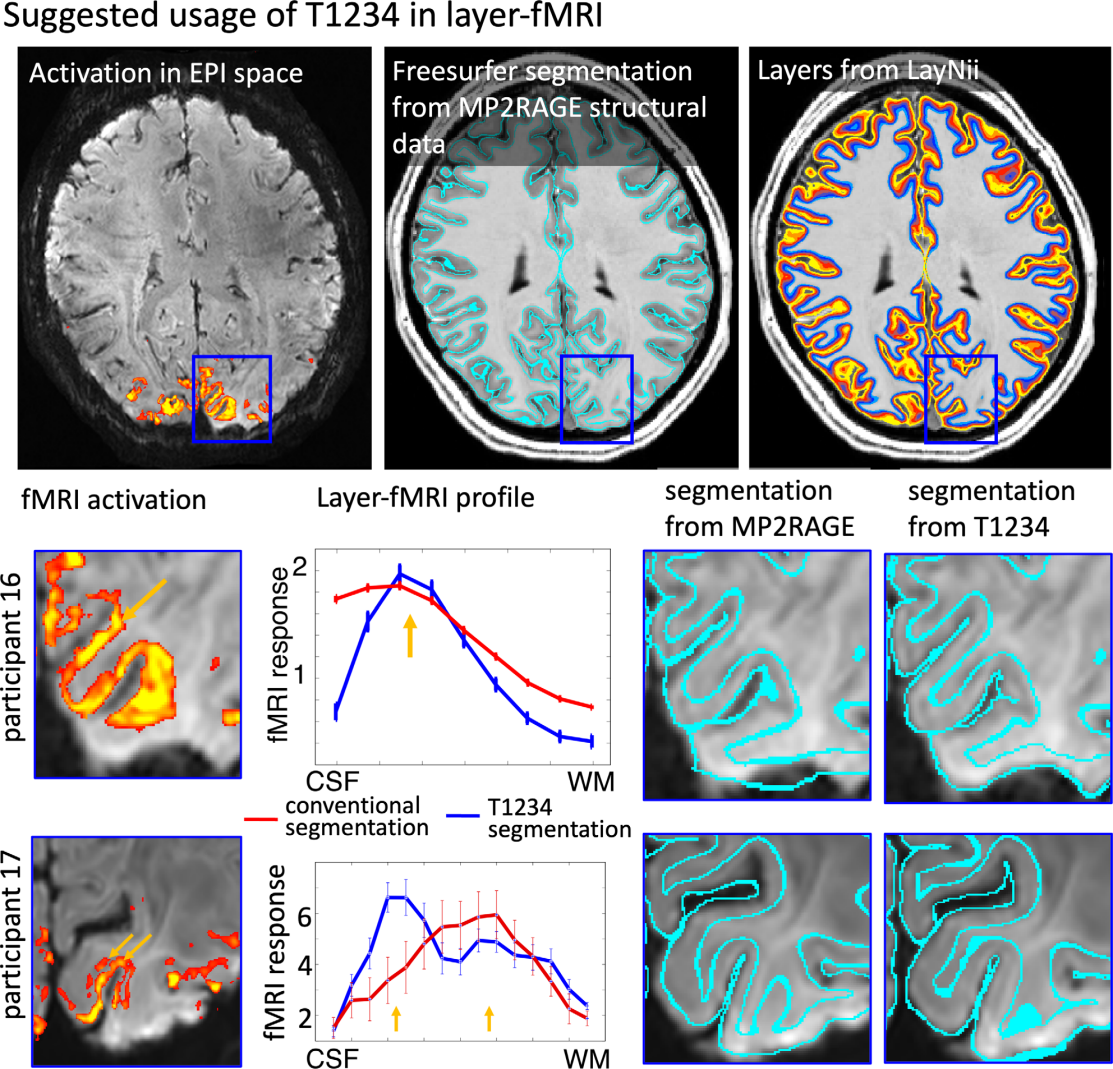
Proposed usage of T1234 for high resolution layer‐fMRI applications. Conventional layer‐fMRI activation mapping typically uses MP2RAGE structural reference data. These structural reference data are segmented in software tools like FreeSurfer to estimate layers for the extraction of activation scores across cortical depth. However, if the alignment between tissue‐type borders (as derived from structural reference data) does not perfectly match the locations of functional activation data, the resulting layer profiles are compromised. The panels for participants 16 and 17 illustrate two challenges of the conventional layer‐mapping approach. The orange arrows point to layer‐specific VASO activation patterns that follow the cortical ribbon at unique cortical depths. However, when these signals are pooled from compromised segmentation borders of conventional structural reference data, the depth‐dependent peaks are blurred. When segmentation borders have higher spatial precision from T1234 data, the peaks of depth‐dependent activations become visible in layer profiles. The turquoise overlay refers to Freesurfer segmentations in voxel‐space (aka rim) as used for layerification in LayNii. T1234 data in this figure were scanned with protocol parameters as described in the “acquisition distortion‐matched approach.” Functional acquisition parameters are included in the protocol “functional_protocol.pdf” in https://github.com/layerfMRI/Sequence_Github/blob/master/T1234 (tag: T1234release).

To demonstrate the value of the distortion‐matched T1234 sequence for layer‐fMRI research, we compared laminar fMRI signal pooling using T1234‐derived tissue borders with signal pooling using conventionally derived MP2RAGE borders. Figure 5 shows that laminar Vascular Space Occupancy (VASO) signal pooling from distortion‐matched T1234‐derived layer segmentation retains spatial signal modulation across cortical depth with higher spatial fidelity.

## 4 DISCUSSION

### 4.1 Importance of high‐precision tissue type segmentation

Current high‐resolution fMRI studies are often constrained by precision of knowing which functional voxels refer to which cortical layer. Inaccuracies can arise from two main sources (1) misregistration of the structural and functional data and (2) inaccuracies in segmentation. The method provided here is aiming to improve the final segmentation quality in functional data by means of mitigating misregistrations. Previous research has reported that manual correction of automatic and semi‐automatic segmentation approaches require extensive labor.^37^, ^38^ This bottleneck in high‐resolution fMRI has been recognized by the ISMRM Brain Function Study Group as the most significant challenge in mesoscale fMRI. Our T1234 method has the potential to overcome this challenge by achieving high precision without the residual constraints and artifacts associated with other EPI‐based structural mapping protocols.

### 4.2 Optimizing this general T1234 protocol for special cases

Our T1234 approach is optimized for applications in layer‐fMRI and whole‐brain coverage. Although this protocol facilitates straightforward use of FreeSurfer pipelines, it may be less efficient for applications focused solely on individual brain areas. For such cases, optimizing the acquisition protocol for higher resolutions and smaller FOV prescriptions, as demonstrated in Figure 3D, might be more efficient. When even faster acquisitions are needed, higher acceleration factors might be preferred as well.

### 4.3 Limitations of universal B_1_ calibration

The protocol used here enables quantitative T_1_ estimation in milliseconds, assuming a universal B_1_^+^ pattern. This method has been effective for B_1_^+^ shimming in previous studies,^39^ but it may not fully account for participant‐specific local deviations from the representative mean. Although this approach is sufficient for anatomical landmark identification, as demonstrated in Figure 3D, applications requiring more precise T_1_ quantification might benefit from acquiring a participant‐specific B_1_ map. This can typically be done in under a minute.

### 4.4 Limitations of EPI

The high segmentation factor proposed in this protocol allows for relatively short echo train lengths of 14 lines per shot, with an image TE of 8 ms. Although this is significantly shorter than conventional EPI readouts, it remains longer than those used in conventional FLASH‐based structural protocols. As a result, the T1234 approach is more susceptible to fast signal decay induced by susceptibility gradients, particularly in brain regions with significant B_0_ inhomogeneity near air cavities. Here, we proposed two independent protocols. The specific acquisition protocol that relies on retrospectively distorting highly segmented acquisitions to match distortions of functional data is less sensitive to such B_0_ issues. However, it must be noted that it requires retrospective distortion estimation and amplification to match the functional space.

Because, EPI‐distortions are dependent on local B_0_‐inhomogeneities, the resulting alignment (and segmentation) contractions are particularly challenged in brain areas close to air cavities. If these areas are of particular interest, fMRI studies might particularly benefit from T1234 protocols.

## 5 CONCLUSIONS

EPI‐based acquisition of structural reference data has the potential to address registration challenges in high‐resolution fMRI studies. However, such approaches have not gained widespread adoption. This is suspected to be because of EPI artifacts associated with functional scanning, which diminish the appeal of using these methods for structural reference. In this study, we propose an EPI readout approach with multiple combinations of read and phase encoding polarities that addresses two critical limitations: distortion and artifact levels. Our protocol offers a fast, whole‐brain scan in just 3 to 4 min and has proven to be robust for tissue type segmentation across different participants and scanners. This sequence is available for sharing on 7 T SIEMENS scanners and is currently being used by over 50 sites worldwide.

## FUNDING INFORMATION

NIH, Grant/Number: NIHMS2059796; MRI scanning was performed in the FMRIF core.

## CONFLICT OF INTEREST STATEMENT

O.F.G. is an employee of Brain Innovation (Maastricht, NL). The work presented here may be partly specific to industrial design choices of SIEMENS Healthineers' UHF scanners. This vendor is used in 83% of all human layer‐fMRI papers (source: www.layerfmri.com/papers).

## ETHICS STATEMENT

MRI data were acquired under the NIH‐IRB (93‐M‐0170, ClinicalTrials.gov: NCT00001360). We thank Shruti Japee for guidance and support with respect to getting privileges for checking pregnancy tests and IRB.

## Data Availability

Data of all participants can be found on Zenodo as two parts: https://doi.org/10.5281/zenodo.13366784 and https://doi.org/10.5281/zenodo.13376563. Analysis scripts are available here: https://github.com/layerfMRI/repository/tree/master/T1234 (tag: T1234release). Scan protocols are available here: https://github.com/layerfMRI/Sequence_Github/tree/master/T1234 (tag: T1234release). The T1234 sequence with on‐scanner reconstruction of coil‐specific dual‐polarity combination can be accessed via the Siemens C2P exchange platform: For European IP addresses https://webclient.eu.api.teamplay.siemens‐healthineers.com/c2p and for United States IP addresses https://webclient.us.api.teamplay.siemens‐healthineers.com/c2p. Search for 3D‐EPI VASO (by Stirnberg and Huber) provided by DZNE.
